# Vaping: Current evidence, what we do and do not know

**DOI:** 10.4102/safp.v68i1.6285

**Published:** 2026-09-30

**Authors:** Febisola I. Ajudua, Emmanuel E. Ajudua

**Affiliations:** 1 Department of Family Medicine and Rural Health, Faculty of Medicine and Health Sciences, Walter Sisulu University, Mthatha, South Africa; 2 Department of Family Medicine and Primary Health Care, Faculty of Health Sciences, Nelson Mandela University, Gqeberha, South Africa

**Keywords:** Vaping, e-cigarettes, e-liquids, tobacco harm reduction, e-cigarette exhaled aerosol residue, electronic nicotine delivery systems, electronic non-nicotine delivery systems, ENDS, ENNDS

## Abstract

Vaping is the inhaling of aerosolised heated liquids in e-cigarettes. It is now known that vaping is not as harmless as was previously described in the mid-2000s when the devices were introduced into the markets across Europe and America. The e-liquids are made of flavourings or psychoactive substances and may or may not contain nicotine. Research shows that these devices and the e-liquids have the potential for causing harm to humans. Although they were marketed originally to assist with breaking addiction to cigarette smoking and tobacco harm reduction, evidence suggests they are not entirely harmless. This continuing medical education article focuses on what is known and what remains unknown about e-cigarettes.

**Contribution:**

This CPD article shows that a lot remains unknown about e-cigarettes and their long-term effects on human health. Also, very little is known about the effect of second-hand exposure to the e-cigarette exhaled aerosol residue. In high-income countries, they are used commonly among adolescents and young adults. In recent years, research in South Africa shows an increasing pattern of use among adolescents and young adults. This raises concerns for potential health risks for this population and the need for research that describes the effectiveness of these devices in tobacco harm reduction and the potential risks of exposure in adolescents and young adults.

## Introduction

Vaping is the inhaling of aerosol (chemicals) from heated liquids using electronic delivery systems. This is a colloquial term used to describe the habit as it involves inhaled vapours (aerosol) from heated liquids in the device. This alternative to cigarette smoking appeared in the mid-2000s and was believed to reduce the risk of inhaling carcinogens and other toxins associated with cigarette smoking.^1^ These devices are often composed of a battery, an atomiser and a cartridge containing liquids called e-liquids, enclosed in a metal container with a mouthpiece (see Figure 1). The most common psychoactive substance in these e-liquids is nicotine, hence the name electronic nicotine delivery systems (ENDS). The alternative without nicotine is called electronic non-nicotine delivery systems (ENNDS) and may contain other psychoactive substances such as cannabis. In some instances, these e-liquids are nicotine-less flavours that were previously marketed as entirely harmless. This assertion and the gap in legislation about marketing these products when they were first introduced to markets were exploited to market the products to vulnerable populations.^2^ The *Tobacco Products Control Amendment Act 63 of 2008* that governs tobacco use in South Africa (SA) predates the era of e-cigarettes. This law directly restricts the sale of tobacco products to minors, prohibits advertising of these products, and includes increased taxation on these products. South Africa has implemented the Framework Convention on Tobacco Control using the MPOWER initiative recommended by the World Health Organization (WHO) (a technical package of tobacco demand reduction).^3^ In 2022, a bill was introduced in the South African parliament, specifically mentioning electronic devices. It has the potential to limit exposure of children and adolescents to the sale of ENDS and ENNDS.^4^

**FIGURE 1:**
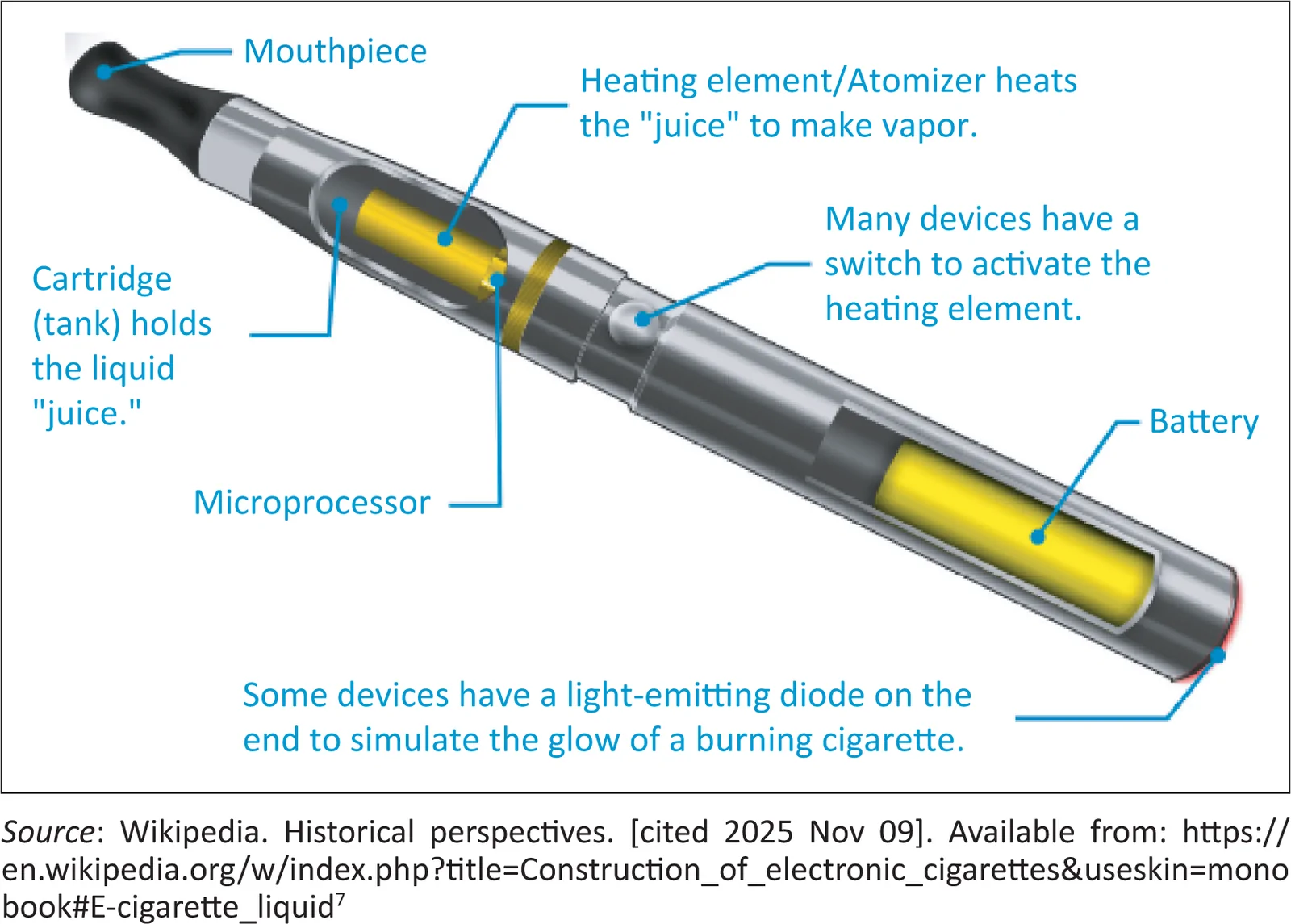
The parts of an e-cigarette.

The electronic cigarette, in its current battery-operated form, was first invented by a Chinese pharmacist, Hon Lik.^5^ His motivation was to develop a device that would help him stop a heavy smoking habit. He had watched his father struggle with lung diseases due to smoking. In the past 100 years, many similar ideas were developed, but the current invention dates to 2003.^6^

The use of ENDS was initially marketed as a less harmful means of tobacco use. It was essentially introduced by tobacco companies as a means for tobacco harm reduction. It was marketed originally to assist persons with nicotine addiction in stopping the habit and was marketed as such, a less harmful means of delivering nicotine to these clients. It was considered a more appealing way to access nicotine and was marketed increasingly not only to adults trying to quit smoking but also to adolescents and young adults who viewed it as less harmful.^8,9^ The public health impact of smoking made this rather easy, as it was perceived as less harmful to the environment and believed not to be associated with secondary smoking. Electronic nicotine delivery systems have since become widely used to access nicotine, a highly addictive substance, and are increasingly used by adolescents and young adults.^10,11^

Although e-cigarettes are generally considered less harmful than traditional cigarettes, two decades after their introduction to international markets, there is growing concern that increasing numbers of young people who have never used tobacco are now taking up these products.^12,13^

## Epidemiology

In preparing this continuing medical education subject, the authors appraised review articles, population surveys, and reports of expert opinions on the effect of e-liquids and e-cigarettes on human health. The trends show use of ENDS among persons seeking to stop harmful use of tobacco products, as well as adolescents and young adults who are curious about the novelty of e-cigarettes and the many flavours of e-liquids.^14,15^ A morbidity and mortality survey report from the United States in 2024 revealed 5.4% of students in middle and high school used e-cigarettes.^11^ It is believed that there is a higher awareness of vaping in these populations than in low- and middle-income country (LMIC) populations.^12^ The findings of a self-administered survey among South African students reported that 16.83% of over 25 000 students across 52 schools were actively vaping. In the same study, more than one-third of participants reported having used the device at some point. Curiosity and social influences were the most common reasons for initiating use, while continued use was mainly attributed to enjoyment and stress relief.^13^ These findings suggest the challenges of increasing exposure among adolescents and young adults who were never cigarette smokers and the potential for ENDS & ENNDS to be a ‘gateway’ to nicotine addiction and potentially other psychoactive substances such as cannabis.

The WHO factsheet flags e-cigarettes as harmful to human health; there is no substantive evidence of their usefulness in combatting nicotine addiction.^16^ The paucity of directed policies to control marketing of these products when they were first introduced to markets left a gap in legislation, allowing these products to be marketed as safe alternatives to smoking. The effect of this gap is that a larger population of consumers of these products started out experimenting and became established users of these products as a gateway to smoking cigarettes.^1^ A telephonic survey conducted in SA in 2022 similarly revealed the potential of these products for helping with tobacco harm reduction but also being a potential gateway to smoking and the use of other tobacco products.^17^ A Cochrane database systematic review published in 2021 included 61 studies (34 randomised controlled trials) and surmised that ENDS were potentially more useful in helping longstanding cigarette smokers quit nicotine addiction when compared to other adjuncts for therapy.^18^

In the early days, there was limited knowledge about the potential impact of these electronic devices on human health, and so the rapid uptake across many countries. A critical umbrella review in 2023 by authors across Europe, the Americas, Australia and North Africa found e-cigarettes are useful in reducing cigarette smoking.^19^ However, another meta-analysis of randomised controlled trials was inconclusive as it included very small population studies which were not powered to give conclusive evidence.^20^ A South African-authored narrative review of South African papers describes a widespread use of e-cigarettes and highlights the benefits of legislation that controls the sale of e-cigarettes similar to other tobacco products.^21^ There is a paucity of studies on the use of e-cigarettes for tobacco harm reduction in the South African population.

In 2016, the WHO published recommendations for tobacco control highlighting emerging reports of the harmful effects of these products and their impact on cardiovascular and respiratory health.^22^ A bill was presented in parliament in 2022 that would address most concerns expressed by clinical experts to restrict access to ENDS and ENNDS like other tobacco products. This bill has not yet been passed.^4^ The legislation in SA does not provide restrictions on the marketing and use of ENDS and ENNDS.

## Effects of e-liquids and parts of the electronic devices on human health

The inhalation of aerosolised liquids containing ultra-fine particles that are potentially toxic is now described through case reports, surveys and toxicology reports.^23,24^ These particles, depending on the type, affect various aspects of the human body. An overview of the effects of e-cigarettes based on human and laboratory studies reveals the potentially toxic nature of heating all components of the e-cigarette^25^ (see Table 1).

**TABLE 1:** Human health effects of exposure to e-cigarette use.

Components of e-cigarettes	Identified chemicals	Health effects
Wick	Alloys – iron, nickel, lead, chromium	Contact dermatitis
Atomiser	Metals may contaminate e-liquid	Renal injury, cardiovascular effects, neurotoxicity
Battery		
E-liquid	Ethyl maltol, ethyl vanillin, vanillin, cinnamaldehyde, menthol, propylene glycol, glycerol, nicotine or other psychoactive substances	Inflammation (lungs and systemic), oxidative stress, endothelial dysfunction

*Source*: Bonner E, Chang Y, Christie E, et al. The chemistry and toxicology of vaping. Pharmacol Ther. 2021;225:107837. https://doi.org/10.1016/j.pharmthera.2021.107837^27^; Farsalinos KE, Voudris V, Poulas K. Are metals emitted from electronic cigarettes a reason for health concern? A risk-assessment analysis of currently available literature. Int J Environ Res Public Health. 2015;12(5):5215–5232. https://doi.org/10.3390/ijerph120505215^33^; Hughes A, Hendrickson RG. An epidemiologic and clinical description of e-cigarette toxicity. Clin Toxicol. 2019;57(4):287–293. https://doi.org/10.1080/15563650.2018.1510503^34^

### Effects on the human lung tissue

It is established that the exposure of lung tissue to vapours of both nicotine-containing and non-nicotine e-liquids and flavours has cytotoxic effects.^26^ These aerosols are said to induce inflammation and oxidative stress, alter cell morphology, lead to cell death and interfere with cell membranes.^27^ These effects are described as similar to what is observed in patients with chronic obstructive airway disease.^28^

In 2019, it was established that vaping and the use of e-cigarettes were associated with acute lung injury called e-cigarette and vaping product-associated lung injury (EVALI).^29^ E-liquids contain solvents generally regarded as non-toxic, most commonly propylene glycol, glycerol and vitamin E acetate. While these substances are considered safe for topical application or ingestion, they become harmful when heated and inhaled, impairing lung immunity and triggering inflammation.^30,31^ See Table 1 for some observed effects of components of the e-cigarette. Toxicologists have described in animal models how heating e-liquids could release potentially toxic aerosol chemicals from these flavours that cause direct injury to the alveolar membrane. Some of the chemicals identified include diacetyl, which has been associated with bronchiolitis obliterans (popcorn lung).^26^ This means that the potential for lung injury is not only due to tobacco or nicotine-containing products but also nicotine-less flavours previously believed to be harmless.

Imaging experts describe the commonly identified patterns of imaging associated with the use of vaping products as consolidations, ground-glass opacities, centrilobular nodules and pleural effusions.^32^ E-cigarette and vaping product-associated lung injury can resemble acute lung infections, leading to initial treatment with broad-spectrum antibiotics. However, patients often benefit from glucocorticoid therapy, and some improve with supportive care alone.^29^ It is essential that patients diagnosed with EVALI discontinue the use of e-cigarettes to prevent further deterioration in lung function. Long-term outcomes are not clear, and this remains an essential gap for further research to describe these outcomes.

### Cardiovascular effects

The aerosol inhaled from these devices contains ultra-fine particles that can trigger inflammation in the lungs and the cardiovascular system. Expert opinion suggests the mechanisms of pathology in the cardiovascular system include oxidative stress, endothelial dysfunction, platelet activation and atherogenesis.^25^ Evidence currently available from clinical experts suggests e-cigarettes are not entirely devoid of harm, as may be believed among users. This should be highlighted in any marketing or advertising allowed for these devices. The long-term impact of these effects should be a subject of further research.

### Second-hand exposure to e-cigarettes

Research models describe how non-users may inadvertently be exposed to ultrafine particles called e-cigarette exhaled aerosol residue (ECEAR). The residue is found on surfaces in rooms or spaces where e-cigarettes have been used.^35^ Studies have not been able to associate second-hand exposure to increased risk for asthma in children but have shown an increase in acute exacerbations among known asthmatics. A systematic review of health effects of second-hand exposure showed that although biomarkers of exposure were increased, this was not associated with health effects.^36^ Another study found that second-hand exposure was associated with increased risk for chronic bronchitis among young adults. It is essential to establish which is accurate through prospective studies that describe health effects of second-hand exposure.

It is also highlighted in expert reviews that not enough information is available at present regarding the long-term effects of e-cigarettes. The implications of heating other components of the e-cigarette are not yet established. Currently, research focuses on the observed effects of heating e-liquids. It is established that the various components have toxic effects, but it is not known how carcinogenic they are. Table 2 compares identified harmful effects of e-cigarettes compared with cigarette smoking.

**TABLE 2:** Potential health effects of e-cigarettes compared with cigarette smoking.

E-cigarette	Cigarette smoking
Chronic obstructive airway disease	Chronic obstructive airway disease
Chronic bronchitis	Emphysema, Chronic bronchitis
Exacerbation of asthma	Exacerbation of asthma
Lipoid pneumonia	Cardiovascular diseases – stroke, peripheral vascular diseases, endothelial dysfunction, cardiac diseases
Popcorn lung	Lung cancer
Interstitial pneumonia	Oral cancer
Lipoid pneumonia	Throat cancer
Organising pneumonia	Cancers in other systems due to active carcinogens in cigarette smoke
Structural lung damage	-
Eosinophilic pneumonia	-
Oral mucosal changes, e.g. periodontal diseases	-
Ultra-fine particles cause direct inflammatory changes in the cardiovascular system	-
Contact dermatitis with prolonged exposure to metal components	-

*Source*: Yayan J, Franke KJ, Biancosino C, Rasche K. Comparative systematic review on the safety of e-cigarettes and conventional cigarettes. Food Chem Toxicol. 2024;185:114507. https://doi.org/10.1016/j.fct.2024.114507^10^; Hughes A, Hendrickson RG. An epidemiologic and clinical description of e-cigarette toxicity. Clin Toxicol. 2019;57(4):287–293. https://doi.org/10.1080/15563650.2018.1510503^34^; Zohara Z, Abdul Jaffar Azad AR. Breaking the myths around e-cigarettes: A narrative review exploring the impact of e-cigarette on human health. Cureus. 2024;16(10):e72039. https://doi.org/10.7759/cureus.72039^37^; Shim TN, Kosztyuova T. Allergic contact dermatitis to electronic cigarette. Dermatitis. 2018;29(2):94–95. https://doi.org/10.1097/DER.0000000000000337^38^

## Conclusion

A lot remains unknown about the health effects of e-cigarettes. Toxicology experts have identified known toxins associated with these devices. Evolving evidence suggests that e-cigarettes are less harmful than the known effects of cigarette smoking. However, not much is known about the long-term effects of e-cigarettes.

Studies in the last two decades have focused on e-liquids because they are aerosolised and in direct contact with human tissue (inhaled or deposited on surfaces as ECEAR). Not much is known about the effects of the materials used in the other components of the e-cigarette. Although they were introduced as devices to reduce the harmful effects of tobacco smoking, they were marketed by manufacturers as less harmful. The widespread use among adolescents and young adults who were not previously cigarette smokers has raised interest in their potential as a ‘gateway’ to using addictive psychoactive substances such as nicotine and cannabis. It is still unknown what the public health implications are for young adolescents gaining access to these devices so early in life and the potential harms in later years. Primary care clinicians should routinely screen for the use of e-cigarettes and related devices, alongside established questions about alcohol, illicit drugs and cigarette smoking. Identifying use enables clinicians to consider potential adverse effects in the differential diagnosis and, when appropriate, refer patients to respiratory medicine specialists who are better equipped to manage these complications.

Research on e-cigarettes for tobacco harm reduction is largely conducted in high-income countries (HICs), with little to none found in LMICs; there is evidence to support their use in breaking nicotine addiction. It is still unclear what pattern of tobacco harm reduction is used across many LMICs for tobacco harm reduction. Studies in South Africa show that many more adolescents use these devices, as described in HICs. Nonetheless, further research is still required to determine the true potential of e-cigarettes in tobacco harm reduction and the long-term effects of use on the health of populations in LMICs.
